# Oxidative Stress and NLRP3-Inflammasome Activity as Significant Drivers of Diabetic Cardiovascular Complications: Therapeutic Implications

**DOI:** 10.3389/fphys.2018.00114

**Published:** 2018-02-20

**Authors:** Arpeeta Sharma, Mitchel Tate, Geetha Mathew, James E. Vince, Rebecca H. Ritchie, Judy B. de Haan

**Affiliations:** ^1^Oxidative Stress Laboratory, Basic Science Domain, Baker Heart and Diabetes Institute, Melbourne, VIC, Australia; ^2^Heart Failure Pharmacology Laboratory, Basic Science Domain, Baker Heart and Diabetes Institute, Melbourne, VIC, Australia; ^3^Cellular Therapies Laboratory, Westmead Hospital, Sydney, NSW, Australia; ^4^Inflammation Division, Walter and Eliza Hall Institute, Melbourne, VIC, Australia; ^5^Department of Medical Biology, University of Melbourne, Melbourne, VIC, Australia

**Keywords:** NLRP3 inflammasome, diabetic nephropathy, diabetic atherosclerosis, diabetic cardiomyopathy, inflammation, inflammatory cytokines, oxidative stress, diabetic complications

## Abstract

It is now increasingly appreciated that inflammation is not limited to the control of pathogens by the host, but rather that sterile inflammation which occurs in the absence of viral or bacterial pathogens, accompanies numerous disease states, none more so than the complications that arise as a result of hyperglycaemia. Individuals with type 1 or type 2 diabetes mellitus (T1D, T2D) are at increased risk of developing cardiac and vascular complications. Glucose and blood pressure lowering therapies have not stopped the advance of these morbidities that often lead to fatal heart attacks and/or stroke. A unifying mechanism of hyperglycemia-induced cellular damage was initially proposed to link elevated blood glucose levels with oxidative stress and the dysregulation of metabolic pathways. Pre-clinical evidence has, in most cases, supported this notion. However, therapeutic strategies to lessen oxidative stress in clinical trials has not proved efficacious, most likely due to indiscriminate targeting by antioxidants such as vitamins. Recent evidence now suggests that oxidative stress is a major driver of inflammation and vice versa, with the latest findings suggesting not only a key role for inflammatory pathways underpinning metabolic and haemodynamic dysfunction in diabetes, but furthermore that these perturbations are driven by activation of the NOD-like receptor family pyrin domain containing 3 (NLRP3) inflammasome. This review will address these latest findings with an aim of highlighting the interconnectivity between oxidative stress, NLRP3 activation and inflammation as it pertains to cardiac and vascular injury sustained by diabetes. Current therapeutic strategies to lessen both oxidative stress and inflammation will be emphasized. This will be placed in the context of improving the burden of these diabetic complications.

## Introduction

Therapeutic strategies to limit diabetic micro and macrovascular complications have focussed first and foremost on eliminating known risk factors. However, despite the availability of numerous drugs such as statins to lower lipids, angiotensin converting enzyme (ACE) inhibitors and angiotensin receptor blockers to control blood pressure, orlistat to reduce weight gain (Wilding, 2018), and a range of insulin-sensitizing drugs such as metformin, sulfonylureas, glitazones, gliptins, α-glucosidase inhibitors, and sodium-glucose transporter-2 inhibitors, for many diabetic patients the inevitable slide toward heart disease, renal failure, neuropathy and diabetic blindness continues unabated, leading to increased morbidity and mortality. In looking further afield at significant drivers of these diabetic complications, it is now increasingly appreciated that inflammatory processes *per se* play a key role, underpinning all of the complications associated with diabetes. Thus, inflammation is now considered a driving force in the progression of diabetic complications and is no longer simply viewed as an epiphenomenon. Indeed, it is now recognized that inflammation is not limited to the control of pathogens by the host, but rather that sterile inflammation which occurs in the absence of viral or bacterial pathogens accompanies the complications that arise as a result of hyperglycaemia.

**Figure 1:**
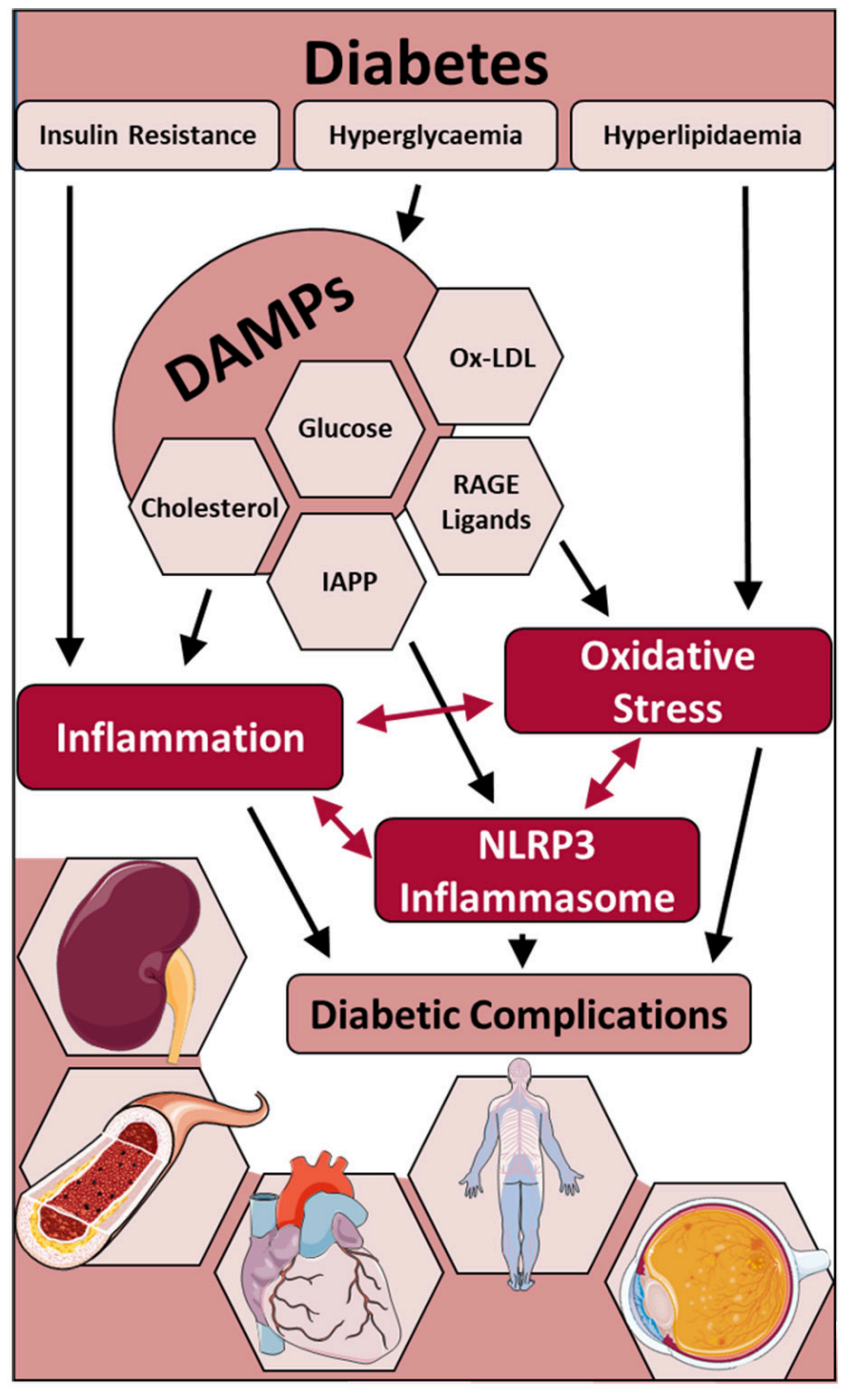
The role of the NLRP3 inflammasome and interconnectivity with oxidative stress and inflammation in the development of complications in diabetes. Metabolic changes in diabetes including insulin resistance, hyperlipidaemia and hyperglycaemia (and the subsequent production of DAMPs), lead to an increase in inflammation, oxidative stress and NLRP3 inflammasome activation and subsequent development of diabetic complications including diabetic nephropathy, atherosclerosis, diabetic cardiomyopathy, diabetic neuropathy and diabetic retinopathy. DAMPs, damage associated molecular patterns; RAGE, receptor for advanced glycation end-products; IAPP, islet amyloid polypeptide protein.

In the years since a unifying mechanism of hyperglycemia-induced cellular damage (Brownlee, 2001) was proposed linking elevated blood glucose levels with oxidative stress and dysregulation of metabolic pathways, pre-clinical evidence has, in most cases, supported this notion. However, therapeutic strategies to lessen oxidative stress in clinical trials have not proved efficacious, most likely due to the indiscriminate targeting by antioxidants such as vitamins. With evidence now suggesting that oxidative stress begets inflammation and vice versa, the latest findings suggest not only a key role for inflammatory pathways underpinning metabolic and haemodynamic dysfunction in diabetes, but furthermore that these perturbations are driven by activation of the NOD-like receptor family pyrin-domain-containing-3 (NLRP3) inflammasome. This review addresses these latest findings with an aim of highlighting the interconnectivity between oxidative stress, NLRP3 activation and inflammation as it pertains to cardiac, vascular and renal injury sustained by diabetes (Figure 1). Current therapeutic strategies to lessen both oxidative stress and inflammation are emphasized, and placed in the context of improving the burden of these diabetic complications.

## Oxidative stress, inflammation and diabetes

Prior to the proposal of the Brownlee unifying mechanism (Brownlee, 2001), research into the pathophysiology of diabetic complications suggested that several important metabolic pathways, namely the polyol, hexosamine biosynthesis, protein kinase C, and the production of advanced glycosylation end-products, were compromised as a result of elevated blood glucose. In understanding how these pathways were affected, it was discovered that the reactive oxygen species (ROS) superoxide (O_2_^.−^), derived from the mitochondria or elsewhere, were capable of damaging a key glycolytic enzyme glyceraldehyde dehydrogenase (GAPDH), which in turn, diverts upstream glycolytic metabolites into the four pathways of glucose overutilization. Subsequent studies have also implicated the NADPH oxidases (NOX) family of enzymes as major cytosolic sources of superoxide, and it is now appreciated that several sources exist within the cell that contribute to the increased oxidative stress accompanying diabetes (de Haan and Cooper, 2011). In addition to the metabolic perturbations, ROS are also known to cause alteration at the molecular level, with numerous reports of enhanced lipid peroxidation, protein modifications, and nucleic acid damage as a consequence of elevated glucose. Thus, it was postulated that newer more targeted antioxidants (such as SOD mimetics or catalase) might offer better protection against ROS damage (de Haan and Cooper, 2011), but in the years since those initial findings, clinical trials with more basic antioxidants (Vitamins C and E) such as HOPE and GISSI have shown no cardiovascular health benefits (Marchioli et al., 2001). More recent antioxidant approaches of note are based on positive pre-clinical data showing a role for specific isoforms of the NOX enzymes in driving diabetic complications (Gray and Jandeleit-Dahm, 2015; Di Marco et al., 2016). Indeed, current trials using specific Nox1/Nox4 inhibitors to limit diabetic nephropathy are ongoing.

One aspect not covered by Brownlee's unifying mechanism (Brownlee, 2001) is the role that ROS play in modulating the signaling cascade of immune factors. It has become apparent that increased ROS production leads to the increased production of inflammatory cytokines, and reciprocally, an increase in inflammatory cytokines can stimulate ROS production. This cyclical process, where oxidative stress begets inflammation and vice versa, drives a highly pro-inflammatory state. Evidence for this stems from the plethora of investigations across numerous diabetic complications, in which the emphasis has shifted away from a purely metabolic state to one that additionally encompasses an inflammatory state (Hotamisligil, 2006; Engelbertsen et al., 2012; Ruiz et al., 2013; Paterniti et al., 2015), thereby modifying the therapeutic approach to now also include drug regimens that target the heightened inflammation.

## Inflammasome activation and regulation

### Inflammasomes activate capase-1 to process IL-1β, IL-18, and trigger cell death

**Figure 2:**
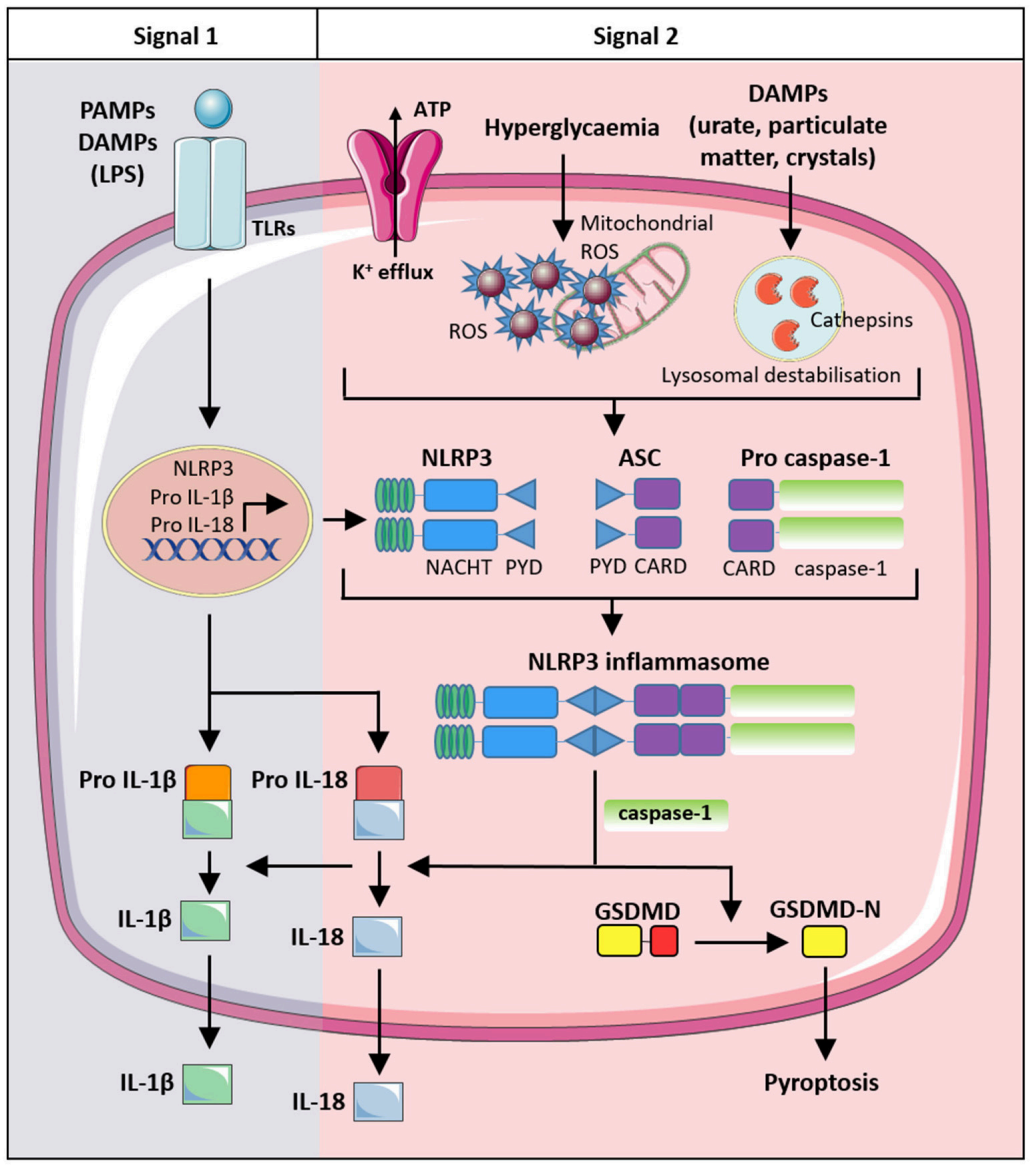
A diagrammatic representation of NLRP3 inflammasome activation. NLRP3 activation is a distinct two-step process requiring a priming (“signal 1”) signal and an activating (“signal 2”) signal. The priming signal is usually initiated by PAMPs (LPS) that bind to toll-like receptors (TLR) and induce expression of NLRP3, pro IL-1β, and pro IL-18. The second activating signal is required for NLRP3 inflammasome assembly, by binding of NLPR3 and pro caspase-1 to adaptor protein ASC. Activating signals include potassium efflux through ion channels and cathepsin release by lysosomal degradation. In settings of diabetes, excessive ROS production in the cytosol and mitochondria has been implicated in NLRP3 inflammasome activation. Following NLRP3 assembly and activation, caspase-1 undergoes proteolytic cleavage and further processes pro IL-1β and pro IL-18 into their mature forms for secretion. In addition, caspase-1 also cleaves GSDMD which results in plasma membrane pore formation and pyroptototic cell death. PAMPs, pathogen associated molecular patterns; DAMPs, damage associated molecular patterns; LPS, lipopolysaccharides; ASC, apoptosis-associated speck-like protein containing a caspase recruitment domain (CARD); GSDMD, gasdermin D; CARD, caspase recruitment domain; PYD, PYRIN domain; NACHT, a domain containing NAIP, CIITA, HET-E and TEP1 domains.

Microbial, host-derived and environmental molecules are all capable of activating large cytosolic protein complexes known as inflammasomes. The ability of inflammasomes to specifically detect cellular stressors, metabolic changes and danger molecules makes them central governors of the innate immune response. Although protective against infections and important for an appropriate immune response following tissue damage, excessive inflammasome activity has now been linked to numerous diseases, including cancer, neurodegenerative disorders, and metabolic dysfunction (Menu and Vince, 2011). NLRP3 activation and subsequent IL-1β secretion is well-characterized in cells of the innate immune system, such as monocytes, macrophages and neutrophils, however, other cell types such as endothelial cells are able to produce IL-1β via the NLRP3 inflammasome (Lopez-Castejon and Brough, 2011; Xiao et al., 2013). Activation of the NLRP3 inflammasome is a two-step process (Signal 1 and Signal 2). In the first step (Signal 1), damage associated molecular pattern (DAMPs) or pathogen associated molecular patterns (PAMPs) activate toll-like receptors (TLRs) to induce the expression of inactive NLRP3, IL-β and IL-18. Subsequently, a second signal, such as bacterial toxins or host metabolites (e.g., ATP), are detected by inflammasome sensor proteins, resulting in their recruitment of the adaptor protein ASC [apoptosis-associated speck-like protein containing a caspase activation and recruitment domain (CARD)] through homotypic pyrin-pyrin domain interactions. A single peri-nuclear oligomerized ASC “speck” subsequently forms in each cell, which is reported to adopt a prion-like structure (Cai X. et al., 2014; Franklin et al., 2014; Lu et al., 2014). Oligomerized ASC binds pro-caspase-1 through homotypic CARD-CARD interactions resulting in proximity-induced caspase-1 processing and activation. Caspase-1 cleaves the inactive precursor proteins of IL-1β and IL-18 into their bioactive fragments that are subsequently released from macrophages into the extracellular milieu. In parallel with caspase-1 cleavage of IL-1β, caspase-1 or the related inflammatory caspase, caspase-11, can cleave gasdermin D (GSDMD)(Kayagaki et al., 2015; Shi J. et al., 2015) thereby triggering an oligomerized N-terminal GSDMD fragment to form pores in the plasma membrane and induce the lytic pro-inflammatory cell death known as pyroptosis (Aglietti et al., 2016; Ding et al., 2016; Liu et al., 2016; Sborgi et al., 2016) (Figure 2). While GSDMD-induced membrane pore formation, and presumably cell death, is important for bioactive IL-1β release from macrophages, other cell types such as neutrophils have been reported to secrete active IL-1β without any compromise in membrane permeability (Chen et al., 2014). This suggests that cell-specific differences, and possibly distinct mechanisms, in IL-1β release exist and that the cellular exit of IL-1β can occur due to an active, yet ill-defined, secretory process (Conos et al., 2016). Alternatively, it has become clear that other cell death modalities that result in a compromised macrophage plasma membrane, such as Mixed Lineage Kinase Domain Like (MLKL) mediated necroptosis, may also cause activated IL-1β release even when GSDMD is genetically deleted (Conos et al., 2017; Gutierrez et al., 2017).

### Excessive inflammasome activation results in autoinflammatory disease

A variety of inflammasome sensor proteins have been identified. These include several members of the NOD-like Receptor family, such as NLRP1, NAIP/NLRC4, NLRP3, NLRP6, NLRP7, and NLRP9 in addition to the HIN-200 family member, AIM2, and the tripartite motif-containing (TRIM) family member Pyrin (also known as TRIM20). Evidence from the literature suggests that several inflammasome sensors have well-defined activating ligands (such as the DNA-binding AIM2) (Latz et al., 2013) and/or mechanisms (such as toxin-induced Rho-GTPase inactivation, which then permits Pyrin to be released from 14-3-3 protein inhibition) (Latz et al., 2013). However, a consensus on precisely how the inflammasome sensor proteins implicated in the metabolic syndrome and diabetic conditions, NLRP1 and NLRP3, come to be activated in these states, has yet to be reached. Regardless, the ability of inflammasomes to trigger damaging autoinflammation is undeniable, as evidenced by the rare hereditary inflammatory diseases resulting from activating mutations in NLRP1, NLRP3, Pyrin, and NLRC4 (Manthiram et al., 2017).

The caspase-1 substrate IL-1β has typically been implicated in inflammasome-driven autoinflammation, and IL-1 blockade has shown remarkable success in the clinic in the treatment of inflammasomopathies. More recently, NLRC4 activating mutations have been documented to result in macrophage activation syndrome (Canna et al., 2014), a condition similar to hemophagocytic lymphohistiocytosis, which are both associated with exacerbated IL-18 levels (Kaplanski, 2018). A pathologically pro-inflammatory role for increased IL-18 is also suggested by findings implicating IL-18 signaling in vascular pathologies (Bhat et al., 2015). Despite these observations, other studies have implicated a protective function for inflammasome-activated IL-18 in obesity and the metabolic syndrome (Netea et al., 2006; Zorrilla et al., 2007; Murphy et al., 2016). In this context, Murphy et al. (Murphy et al., 2016) recently identified that IL-18 production is driven by activation of the inflammasome sensor NLRP1, and that NLRP1-induced IL-18 maturation protected against an obese phenotype in mice by stimulating lipolysis. The cell types in which NLRP1 functions to cleave IL-18 remains to be determined although production of IL-18 appeared to be local rather than systemic and most likely occurred within the adipose tissue (Murphy et al., 2016). The extent to which inflammasome and caspase-1 driven GSDMD activity participates in autoinflammatory disease, to either promote IL-1β or IL-18 release or trigger pyroptosis, remains to be clarified.

### Mechanism of NLRP1 and NLRP3 activation

#### NLRP1

Studies suggest that NLRP1 and NLRP3 activity levels alter metabolic homeostasis and can thereby contribute to glucose intolerance and insulin resistance (Stienstra et al., 2010, 2011; Vandanmagsar et al., 2011; Wen et al., 2011; Youm et al., 2011; Murphy et al., 2016). As discussed above, murine studies have documented how the deletion of NLRP1 results in an obese phenotype with increased lipid accumulation and glucose intolerance, and that the NLRP1 deficient phenotype is exacerbated further when animals are placed on a high fat diet (Murphy et al., 2016). Conversely, the same study reported reduced fat mass and metabolic dysfunction in an autoactivating NLRP1 mutant mouse, which was linked to excessive IL-18 activation. Overall, these findings suggest that the NLRP1-Caspase-1-IL-18 axis represents an important metabolic rheostat, and that the elevated IL-18 levels observed in the metabolic syndrome, obese people or type 2 diabetes (Fischer et al., 2005; Hung et al., 2005; Zirlik et al., 2007) may result from an attempt to counteract metabolic dysfunction and insulin resistance. How NLRP1 is activated in the context of excess energy intake remains unknown. However, recent findings from the study of autoactivating NLRP1 mutations suggest that autolytic cleavage of NLRP1 is an important step, removing the inhibitory pyrin domain to allow interactions of its C-terminal CARD domain with either ASC or Caspase-1(Yu et al., 2018).

#### NLRP3

Pathological NLRP3 inflammasome signaling and IL-1β processing has been implicated in widespread conditions beyond diabetes and heart disease, such as gout, cancer and Alzheimer's disease (Guo et al., 2015). It therefore comes as no surprise that NLRP3 activity is normally kept tightly in-check by a number of molecular mechanisms, including both transcriptional and post-translational regulatory events, such as phosphorylation, ubiquitylation, S-nitrosylation, and cleavage (Baker et al., 2017). Defining the precise roles and timing of many of these modifications, and how they impact and co-ordinate with reported modifications of ASC or IL-1β itself, remains a substantial challenge.

A diverse number of molecules implicated in inflammatory conditions, such uric acid crystals associated with gout (Martinon et al., 2006), increased lipids [palmitic acid (Abderrazak et al., 2015), ceramide (Vandanmagsar et al., 2011), cholesterol crystals (Duewell et al., 2010)] and glucose or islet amyloid polypeptide (IAPP) (Masters et al., 2010) associated with the metabolic syndrome, atherosclerosis and type 2 diabetes, have been reported to activate NLRP3. Consistent with this, NLRP3 deficiency or IL-1 inhibition is beneficial in mouse models of these diseases and type 2 diabetic patients treated with anti-IL-1 therapy display improvements in glucose control and markers of systemic inflammation (Esser et al., 2014).

Although numerous pathways leading to NLRP3 signaling by diverse chemical and structural stimuli have been proposed, such as ROS production and lysosomal rupture, the unifying molecular switch that triggers NLRP3 inflammasome assembly remains to be clarified (Lawlor and Vince, 2014). However, despite significant controversy in this area, in recent years there has been a growing consensus that the common determinant dictating NLRP3 activation in nearly all circumstances are levels of intracellular potassium. This stance is consistent with the fact that almost all known NLRP3 activators result in decreased levels of intracellular potassium, and if potassium release is prevented, then NLRP3 activation is inhibited (Pétrilli et al., 2007; Muñoz-Planillo et al., 2013). Similarly, media depleted of potassium to promote potassium efflux suffices to trigger spontaneous NLRP3 activation. NEK7 was recently identified as binding to NLRP3 upon potassium efflux to induce inflammasome formation (He et al., 2016; Schmid-Burgk et al., 2016; Shi et al., 2016), although how decreases in cellular potassium trigger this event is not known. Regardless, potassium efflux invariably occurs following membrane damage. This is likely to explain why NLRP3 is activated by genetically-distinct programmed cell death pathways that all cause membrane damage (Vince and Silke, 2016), and importantly, may also account for why the diverse number of cellular stressors linked to atherosclerosis and diabetes all cause pathological NLRP3 activity.

In summary, it is likely that the two inflammasome sensors, NLRP1 and NLRP3, exert opposite effects in obesity; NLRP1 cleavage of IL-18 acts to limit metabolic dysfunction, while NLRP3 activation of IL-1β is detrimental and promotes glucose intolerance. Given that both the NLRP3 and NLRP1 inflammasome produce mature caspase-1 to cleave IL-18 and IL-1β, how these opposing effects occur, and in which cellular compartments, to drive or limit obesity remain unanswered questions that will be important to resolve.

## Inflammasomes in diabetic complications

### Atherosclerosis

Atherosclerosis is a multi-factorial disease of the large arteries characterized by the deposition and accumulation of lipids and inflammatory cells. It is the underlying cause of life-threatening cardiovascular complications including myocardial infarction and stroke. Diabetes is a prominent risk factor for atherosclerosis with diabetic patients demonstrating a 2 to 4-fold increased incidence in the development of atherosclerosis than the non-diabetic population (Khaleeli et al., 2001).

**Figure 3:**
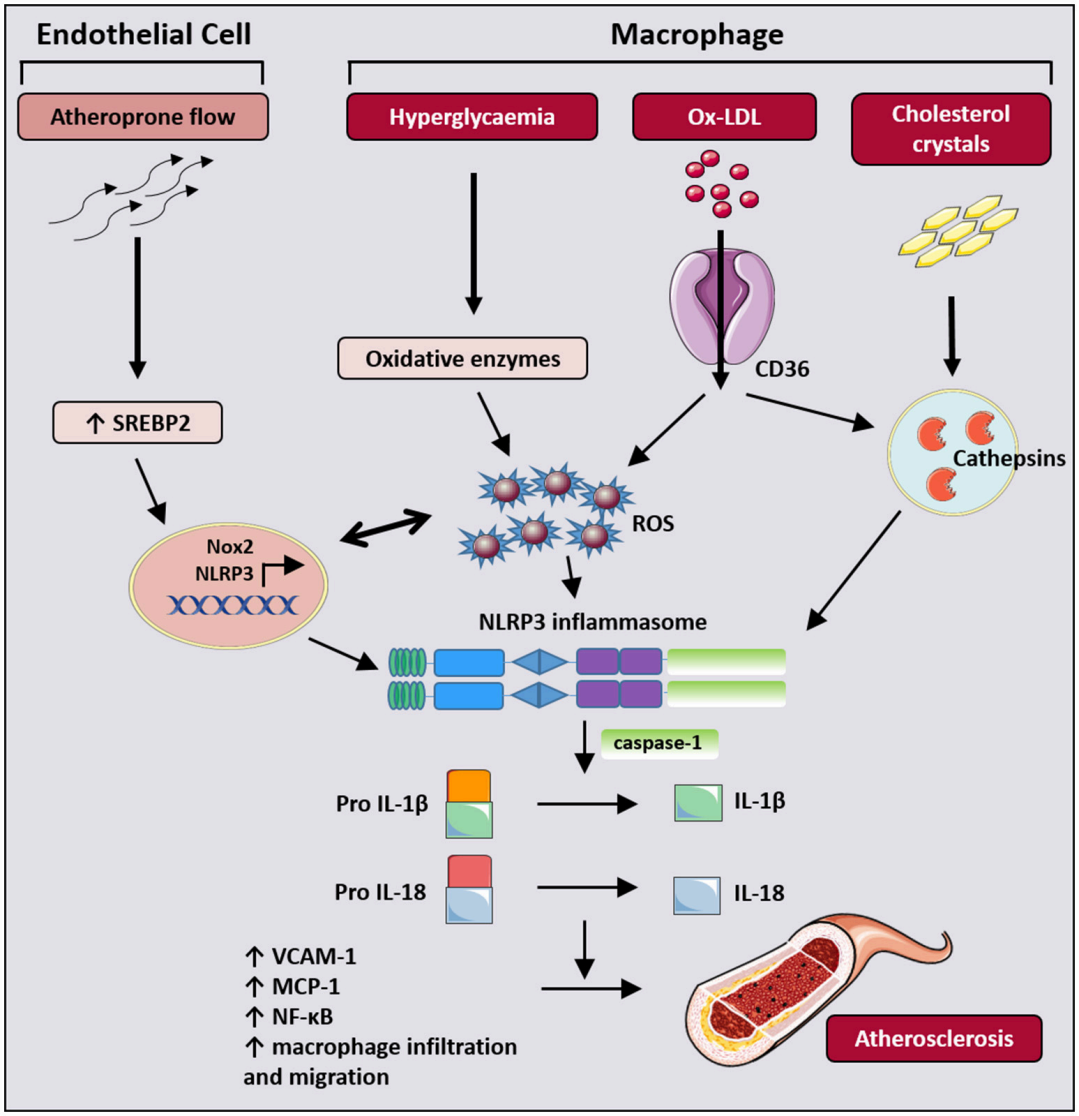
Graphic summary of NLRP3 activation to promote atherogenesis. In endothelial cells, atheroprone or disturbed flow activates sterol regulatory element binding protein 2 (SREBP2) which induces expression of the ROS producing enzyme Nox2 and NLRP3. In macrophages, NLRP3 activation is mediated by the increased presence of glucose-mediated ROS, ox-LDL and cholesterol crystals in diabetic and atherogenic settings. The resultant secretion of IL-1β and IL-18 by both cell types in turn contribute to endothelial dysfunction, macrophage recruitment, migration and activation, and the upregulation of inflammatory mediators including VCAM-1, MCP-1, and NF-κB, leading to progression of atherosclerosis.

A plethora of information, resulting from the analysis of rodent and human atherosclerotic plaques, has demonstrated that IL-1β and IL-18, both of which are products of the NLRP3 inflammasome activation, play a key role in the initiation and progression of atherosclerosis. Deficiency in IL-1β or IL-18 as well as the delivery of antagonist to the IL-1 receptor, has demonstrated marked reduction in atherosclerotic lesion size (Elhage et al., 2003; Kirii et al., 2003; Rader, 2012). Mechanistically, in early and advanced atherosclerotic lesions, the presence of cholesterol crystals was shown to act as the endogenous “danger signal” for the release of cytokines from the inflammasome pathway (Figure 3). This has now been confirmed *in vitro*, in lipopolysaccharide (LPS)-primed peripheral blood mononuclear cells and macrophages exposed to increasing concentrations of cholesterol crystals (Duewell et al., 2010). The release of IL-1β from LPS-primed macrophages subjected to exposure to cholesterol crystals was diminished in macrophages isolated from NLRP3-deficient and ASC-deficient mice, clearly suggesting that cholesterol crystals can act at least in part via the inflammasome pathway to stimulate the inflammatory response (Duewell et al., 2010). Injection of cholesterol crystals results in a state of acute inflammation (assessed by the intraperitoneal accumulation of neutrophils in wild-type mice); genetic deletion of components proposed to activate NLRP3, such as cathepsin B and cathepsin L, or loss of NLRP3 itself, protects mice against this acute inflammation (Duewell et al., 2010). Futhermore, lethally-irradiated low density lipoprotein receptor (LDLR) –/– mice reconstituted with bone marrow from either NLRP3-, ASC-, IL-1α/β or caspase 1/11-deficient mice are protected from the development of diet-induced atherosclerosis (Duewell et al., 2010; Hendrikx et al., 2015), further strengthening the role of myeloid-cell derived NLRP3, ASC, caspase-1 and IL-1β in the pathogenesis of atherosclerosis. Interestingly, examination of the role of NLRP3 inflammasomes in the ApoE KO mouse model of atherosclerosis has however yielded contradictory results. The initial study by Menu et al, demonstrated no differences in atherosclerotic progression, macrophage infiltration in plaques and plaque stability in NLRP3/ApoE, ASC/ApoE and caspase-1/ApoE double KO mice, with the authors concluding that NLRP3 inflammasome activation is not a critical factor in atherogenesis (Menu et al., 2011). Following this, two other studies subsequently reported that caspase-1 deficiency in the high-fat-diet-fed ApoE KO mouse conferred atheroprotection (Gage et al., 2012; Usui et al., 2015). The underlying causes for these discrepancies between the three studies remain unclear, however differences in experimental design, in microbiota environment, and in formulation of the diet (at the level of cholesterol content) might have contributed to the differences in atherosclerosis observed.

Endothelial cells and macrophages are considered the primary cell types that participate in development and progression of atherosclerosis. Recently, several other molecular mechanisms by which NLRP3 inflammasomes contribute to atherogenesis in both cell types have been elucidated. Firstly, the oxidized form of low-density lipoprotein (ox-LDL), a prominent molecule implicated in atherosclerotic plaque development, was shown to play a direct role in NLRP3 activation (Figure 3) and NLRP3-induced macrophage pyroptosis. It was proposed that ox-LDL promoted robust ROS formation and direct ROS-driven activation of NLRP3 and caspase-1, leading to pyroptosis of macrophages and atherosclerotic lesion instability (Lin et al., 2013). Secondly, sterol regulatory element binding protein-2 (SREBP 2), a master regulator of cholesterol biosynthesis, plays a critical role in athero-prone flow-mediated endothelial inflammation via the activation of the NLRP3 inflammasome in endothelial cells. (Li et al., 2013; Xiao et al., 2013). Athero-prone flow activated the endothelium and resulted in the upregulation of SREBP 2, which then induced the transcription of NADPH oxidase 2 (Nox2) and NLRP3 expression, thereby leading to IL-1β expression and endothelial inflammation (Xiao et al., 2013). SREBP-inflammasome-endothelial innate immune responses provide a novel link between endothelial activation and monocyte recruitment, which forms the key process that causes the imbalance in vascular homeostasis, thus leading to atherosclerosis (Figure 3). In addition, this pivotal data provides evidence that endothelial cells play an important role in inflammatory responses through activation of NLRP3 inflammasomes.

In the context of diabetes, the role of inflammasomes in diabetes-associated atherosclerosis has attracted only modest attention. The evidence that has emerged however favors a role for inflammasome-derived cytokine production in this setting. In a short-term model of diabetes-induced endothelial dysfunction, treatment with the IL-1 receptor antagonist, anakinra, restored endothelial-dependent relaxation, with concomitant reduction of vascular NADPH oxidase and NF-κB activation (Vallejo et al., 2014). Moreover, several inflammasome components NLRP3, ASC and IL-1β are elevated at the gene expression level, in a diabetic porcine model of atherosclerosis (Li et al., 2013). In streptozotocin (STZ)-induced diabetic ApoE KO mice, aortic protein expression of NLRP3, ASC, caspase-1, IL-1β, and IL-18 was upregulated in comparison to non-diabetic controls, and was associated with enhanced lesion development and an elevation of ROS levels (Leng et al., 2016). This was confirmed *in vitro* in isolated bone marrow-derived macrophages, which demonstrated increased protein expression of NLRP3 and secretion of IL-1β following stimulation with high glucose and in the presence of LPS (Leng et al., 2016). Thioredoxin-interacting protein (TXNIP), a redox signaling regulator, is upregulated significantly in response to hyperglycemia and is reported to be a direct ligand of the NLRP3 inflammasome, at least in some contexts, although conflicting observations have been reported (Masters et al., 2010). In type 2 diabetes, islet amyloid polypeptide protein (IAPP), a protein responsible for the deposition of amyloid in the pancreas, has also been identified as a trigger for the NLRP3 inflammasome with resultant mature IL-1β secretion from pancreatic islets, thereby further contributing to the inflammatory response in diabetes (Masters et al., 2010). Saturated free fatty acids, for example palmitate, elevated in obese diabetic patients, induces the activation of hematopoietic NLRP3 inflammasomes via a ROS-mediated pathway, ultimately leading to the impairment of insulin signaling in target tissues to reduce glucose tolerance and insulin sensitivity (Lee et al., 2013). However, whilst there has been identification of endogenous “danger signals” that can trigger the inflammasome machinery, their direct link to diabetes-induced macrovascular complications needs further attention.

In the clinical setting, the protein and gene expression of NLRP3, ASC, caspase-1, IL-1β, and IL-18 was significantly increased in unstable carotid atherosclerotic plaques as compared to stable plaques and control patients that had no evidence of coronary artery stenosis (Shi X. et al., 2015; Paramel Varghese et al., 2016). This correlated with increased serum IL-1β and IL-18 from the same patient cohort. Moreover, immunohistochemical localization of NLRP3 has been observed in the cytoplasm of macrophages and foam cells, and associated with cholesterol crystal clefts inside and outside of the cell (Shi X. et al., 2015). Furthermore, polymorphisms in the NLRP3 gene are strongly correlated with an increased risk of macrovascular complications, in particular myocardial infarction, in Type 2 diabetic patients (Klen et al., 2015). Clinical verification of the role of NLRP3 and products of NLRP3 activation in the development of macrovascular complications may serve as a basis to incorporate this inflammasome family, as useful clinical predictors of cardiovascular events, in diabetic patients.

### Diabetic cardiomyopathy

It is becoming increasingly apparent that, in addition to an increase in ROS, inflammasome activation is important in the pathogenesis of type 2 diabetes-mediated cardiomyopathy. Diabetic patients develop a distinct form of cardiomyopathy (Rubler et al., 1972), termed diabetic cardiomyopathy, characterized by structural and functional alterations of the heart (Tate et al., 2017). NLRP3-inflammasome activation and its role in the progression of heart failure in the absence of diabetes is well-described (Butts et al., 2015; Turner, 2016). Indeed, in the setting of acute experimental myocardial infarction, NLRP3 deletion or pharmacological inhibition reduced infarct size (Mezzaroma et al., 2011; Marchetti et al., 2014), whilst activation of NLRP3 led to inflammasome hyperactivation and amplified cardiac injury (Mezzaroma et al., 2011).

Recent reports reveal that inflammasome activation is also crucial in the setting of diabetes-induced cardiomyopathy, with archetypic metabolic disturbances exacerbating diabetes-induced cardiac dysfunction. Both type 1 and type 2 diabetes mellitus are associated with oxidative and nitrosative stress, insulin resistance and deficiency, leading to low-grade inflammation in several tissues (Singh, 2014). The healthy heart consumes a huge amount of energy that is derived from mitochondrial oxidative phosphorylation of fatty acids (~70%) and glucose oxidation (~30%)(Bayeva et al., 2013; Jia et al., 2018). However, in diabetes there is shift toward fatty acid metabolism that is cardiotoxic and contributes toward cardiomyopathy. Recently, the activation of the NLRP3 inflammasome was reported to be associated with this process, whereby NLRP3 activation accompanies increased CD36 expression. CD36 is responsible for the uptake of fatty acids in the heart, whereas glucose uptake is primarily mediated via insulin-dependent glucose transporter, GLUT4. In the healthy heart, nutrients increase plasma insulin levels triggering myocardial insulin signaling and the presence of both GLUT4 and CD36 at the myocyte sarcolemma, supporting metabolic flexibility (Jia et al., 2016). However, when insulin resistance is present, as seen in type 2 diabetes patients, GLUT4 is preferentially internalized, favoring CD36-mediated fatty acid oxidation and metabolic instability, thereby ultimately leading to a state of decreased cardiac efficiency (Sheedy et al., 2013; Jia et al., 2016; Shah and Brownlee, 2016).

NLRP3 is expressed in the key effector cell types in the diabetic heart, namely cardiomyocytes, fibroblasts and leukocytes (Lee et al., 2013; Bracey et al., 2014; Ruscitti et al., 2015; Monnerat et al., 2016). In untreated type 2 diabetic patients, monocytes exhibited increased levels of NLRP3, ASC and pro-inflammatory cytokines (Lee et al., 2013). Monocytes isolated from these patients were more susceptible to damage associated molecular patterns (DAMPs) (Turner, 2016) and caspase-1 cleavage (Lee et al., 2013). DAMPs are molecular signals from the damaged myocardium that include, but are not limited to intracellular molecules not normally accessible to the immune system, cytokines released from damaged cells and ECM degradation products (Turner, 2016). These effects were shown to be mitochondrial ROS- and AMPK-dependent, and interestingly, 2 months of metformin treatment, a known AMPK activator, blunted this pro-inflammatory response (Li et al., 2013). Furthermore, macrophage-dependent IL-1β increased the propensity to develop cardiac arrhythmias in diabetic mice, an effect attenuated by NLRP3 inflammasome blockade and IL-1 receptor inhibition (Monnerat et al., 2016).

As mentioned, the diabetic heart is particularly susceptible to extracellular matrix remodeling. It is now becoming apparent that excessive NLRP3 inflammasome activation is partly responsible for driving the structural and functional changes in the diabetic heart, promoting cardiac inflammation, apoptosis, and fibrosis (Cai J. et al., 2014; Luo et al., 2017). Luo et al. demonstrated that the NLRP3 inflammasome plays a direct role in the progression of diabetic cardiomyopathy (Luo et al., 2014a). Moreover, subsequent activation of IL-1β and caspase-1 initiates cell death via pyroptosis, an effect attenuated by NLRP3 gene silencing (Luo et al., 2014a). Furthermore, Rosuvastatin, a 3-hydroxy-3- methylglutaryl coenzyme A reductase inhibitor shown to have anti-inflammatory and anti-oxidant properties in several disease pathologies (Sharma et al., 2011; Zhang et al., 2012), conferred improvements in the setting of diabetic cardiomyopathy, via an NLRP3 inflammasome-dependent mechanism (Luo et al., 2014b). These beneficial effects were associated with suppressed MAPK signaling. Indeed, the suppression of MAPK signaling is of significance as these pathways are triggered by hyperglycaemia and known to accelerate the development of cardiac fibrosis (Van Linthout et al., 2007; Rajesh et al., 2012).

Cardiac fibroblasts differentiate into myofibroblasts in order to aid wound healing following stimulation primarily by TGF-β and angiotensin II (van den Borne et al., 2010). However, in certain pathologies, including diabetic cardiomyopathy, chronic activation leads to excessive collagen deposition, tissue fibrosis and detrimental cardiac remodeling (Petrov et al., 2002; Tate et al., 2016; Turner, 2016). NLRP3 expression is increased in cardiac fibroblasts following TGF-β stimulation, with NLRP3-deficient cells exhibiting impaired differentiation and Smad signaling (Bracey et al., 2014). Furthermore, NLRP3 inflammasome activation suppressed cAMP release in cardiac fibroblasts and inhibited cardiac contraction (Zhang et al., 2012). The response to tissue damage seems to be a highly-orchestrated response that is temporal in nature and includes all of the key effector cell types, which express NLRP3 and associated proteins. Although no studies to date have investigated fibroblast-to-myofibroblast differentiation in the setting of diabetic cardiomyopathy, it remains possible that the NLRP3 axis may represent a useful therapeutic target to limit this process.

### Diabetic nephropathy

Inflammation is now recognized as an important causal factor of diabetic nephropathy (Wada and Makino, 2013). Understanding which inflammatory pathways are of significance has identified molecules that contribute to disease progression including various transcription factors, pro-inflammatory cytokines, chemokines, adhesion molecules, Toll-like receptors, adipokines and nuclear receptors. Of significance, inflammatory mediators produced by the NLRP3 inflammasome are implicated in diabetic nephropathy (DN). For example, in a recent study by Fu et al., temporal increases in the expression of NLRP3-related proteins (IL-1β, NLRP3, caspase-1) in rats with DN, and in human glomerular mesangial cells under high glucose conditions, were reported (Fu et al., 2017).

The exact mechanism by which the inflammasome is activated in DN is still unclear. Inflammasome activation through the ROS/TXNIP pathway has been reported in glomerular mesangial cells exposed to high-glucose and lipopolysaccharide (Feng et al., 2016). Long coding RNAs (lncRNAs) are also known to play important roles in several diseases. In a recent study by Yi et al. to evaluate the role of lncRNAs in DN, long intergenic noncoding RNA (lincRNA)-Gm4419 was found to be involved in inflammation and fibrosis in mesangial cells exposed to high-glucose through the NFκB/NLRP3 inflammasome signaling pathway (Yi et al., 2017). Glomerular apoptosis mediated by caspase-1-dependent inflammasome activation can also lead to DN (Shahzad et al., 2016a). Mitochondrial ROS is yet another potential mediator which activates the inflammasome in the diabetic milleu leading to DN (Shahzad et al., 2015). In a study to investigate the role of hyperuricemia in DN, Kim et al. reported that uric acid can stimulate the NLRP3 inflammasome in murine macrophages, leading to pro-inflammatory signaling in proximal tubular cells and thereby contributing to DN (Kim et al., 2015).

In DN, treatment with the DPP-4 inhibitor saxagliptin attenuated diabetes-induced activation of the inflammasome and delayed the progression of diabetic nephropathy in experimental models (Birnbaum et al., 2016). In fact, saxagliptin attenuated protein levels of ASC, NLRP3, TNFα, and caspase-1 in renal and adipose tissue (Birnbaum et al., 2016). Interestingly, the effects of saxagliptin on the NLRP3 inflammasome components and subsequent kidney remodeling were comparable in both type 1 and type 2 murine models of diabetes. This occurred despite saxagliptin having no effect on glycaemic control in type 1 diabetes, thereby inferring that the beneficial actions of saxagliptin may, at least in part, be independent of its established glucose lowering actions (Birnbaum et al., 2016).

Recently, Wang S. et al. (2017) reported that IL-22 gene therapy ameliorated renal damage, mesangial expansion and improved renal function in a mouse model of STZ-induced experimental DN. Interestingly, IL-22 therapy inhibited NLRP3 activation, caspase-1 cleavage and IL-1β maturation suggesting an anti-inflammatory action mediated through NLRP3 suppression in the kidney (Wang S. et al., 2017). Furthermore, IL-22 dose-dependently reduced glucose-induced activation of NLRP3 inflammasome in renal mesangial cells suggesting an action independent of improved glycemic control (Wang S. et al., 2017). The glucose-lowering thiazolinedione, pioglitazone, was recently shown to ameliorate glomerular NLRP3 inflammasome activation in diabetic ApoE KO mice. Pioglitazone reduced the expression of AGEs, RAGE, and NFκB, leading to reduced NLRP3 and downstream pro-inflammatory mediators (Wang Y. et al., 2017).

Recent data now suggests a role for the redox-sensitive transcription factor nuclear erythroid 2-related factor 2 (Nrf2) in the protection against DN via inhibition of the NLRP3 inflammasome. The tetracycline antibiotic minocycline is now thought to afford anti-inflammatory effects in diabetic patients, and renoprotection in animal models of DN, via the Nrf2/NLRP3 axis (Shahzad et al., 2016b). Minocycline enhances Nrf2 levels by inhibiting Nrf2 ubiquitination (Shahzad et al., 2016b). Furthermore, the minocycline-mediated NLRP3 inflammasome inhibition and its subsequent therapeutic effect in DN was absent in diabetic Nrf2 KO mice (Shahzad et al., 2016b), thereby implicating Nrf2 protection at the level of the inflammasome. In addition, curcumin, a known Nrf2 activator, ameliorated endpoints of DN and improved kidney function by suppressing NLPR3 signaling in the db/db type 2 diabetic mouse model (Lu et al., 2017). Hence, given the emerging importance of the NLRP3 inflammasome in DN, as evidenced by the above studies, targeting the NLRP3 inflammasome may be a potential therapeutic strategy to effectively treat DN (Sakai and Wada, 2015).

## New therapies that target the inflammasome

In the recent years there has been a paradigm shift in our understanding of inflammatory diseases. Dysregulation of inflammasome activity has been heavily implicated in autoimmune disorders, such as rheumatoid arthritis and cryopyrin-associated autoinflammatory syndrome (CAPS), which led to the development of several IL-1 and IL-18 decoy binding proteins and neutralizing antibodies, such as anakinra and canakinumab (Guo et al., 2015). However, it is becoming more apparent that the inflammasome-driven inflammation is also implicated in sterile inflammatory diseases, such as diabetes, metabolic syndrome, Alzheimer's disease and atherosclerosis, and that there is a need for therapies specifically targeting the mechanistic pathway of inflammasome activation to enhance clinical success. Current therapeutics that target the inflammasome include compounds such as β-hydroxybutyrate (BHB), MCC950, as well as caspase-1 and ASC inhibitors (Guo et al., 2015; Ozaki et al., 2015). We will review drugs/compounds that were originally developed for different indications, but have shown promise at modulating the inflammasome pathway *in vivo* and *in vitro*, as well as the latest results from the Canakinumab Antiinflammatory Thrombosis Outcome Study (CANTOS) trial.

### SGLT2 inhibitors

In type 2 diabetic patients at high risk of experiencing cardiovascular events, treatment with sodium–glucose cotransporter 2 (SGLT2) inhibitors, a new class of glucose-lowering agent, led to a lower rate of death from all cardiovascular causes in the EMPA-REG OUTCOMES trial (Zinman et al., 2015). SGLT2 inhibitors are considered to primarily act by decreasing renal glucose reabsorption via the early proximal tubules of the kidney, subsequently increasing urinary glucose excretion. Interestingly, they also have anti-inflammatory actions and repress the advancement of diabetic nephropathy (De Nicola et al., 2014). In experimental models of both type 1 and type 2 diabetes, treatment with ipragliflozin reduced levels of oxidative stress biomarkers and inflammatory markers in liver and plasma (Tahara et al., 2013), further supporting the premise that SGLT2 inhibition not only improves hyperglycaemia but also diabetes-associated metabolic and inflammatory abnormalities. Ye et al. (2017) were the first to report *in vivo* that SGLT-2 inhibition with a known inhibitor, dapagliflozin, supressed the structural and functional changes that occur in diabetic cardiomyopathy. The findings of this study provided partial mechanistic insight into the encouraging results of the EMPA-REG OUTCOMES trial (Zinman et al., 2015). Superficially, the clinical and experimental findings are somewhat surprising as SGLT2 is not expressed in the heart (Vrhovac et al., 2015), however the *in vivo* dapagloflozin findings were replicated in LPS-induced inflammasome activation studies in isolated cardiomyocytes (Ye et al., 2017), suggesting the beneficial effects are SGLT2-independent. Further interrogation of off-target mechanisms of the SGLT2 inhibitors may yield further mechanistic insights into the net protective actions of this new class of glucose-lowering therapy. For example, the inflammatory effects of dapagliflozin were shown to be at least in part AMPK-dependent (Ye et al., 2017), correlating with findings in other organs that AMPK activation suppresses the upregulation of the inflammasome (Lee et al., 2013; Bae et al., 2016).

Treatment of high glucose-stimulated human proximal tubular cells with empagliflozin supressed the expression of inflammatory and fibrotic markers, seemingly by blocking glucose entry into the cell (Panchapakesan et al., 2013). Furthermore, empaglifozin treatment in high fat fed mice reduced renal tubular damage and attenuated cardiac lipid accumulation, and this was associated with a decrease in NLRP3 inflammasome activation in the kidney (Benetti et al., 2016). Notably, in this study NLRP3 inflammasome activation in the heart was not increased in high fat fed mice after treatment (Benetti et al., 2016). In the vasculature, the SGLT2 inhibitor, dapagliflozin, lessened atherosclerotic lesions in the diabetic ApoE KO mouse, specifically reduced macrophage infiltration in the plaque and enhanced the stability of lesion (Leng et al., 2016). Mechanistically, this was correlated with lowered serum levels of IL-1β and IL-18, NLRP3 protein, and mitochondrial ROS in aortic tissue. Interestingly, inhibition of the NLRP3 pathway was not observed in isolated macrophages treated with dapagliflozin (Leng et al., 2016). Therefore, the exact signaling mechanism that elicits the inhibition of components of the NLRP3 inflammasome by SGLT2 inhibitors may be tissue-specific and requires further evaluation. Nevertheless, these findings clearly provide a rationale to further study SGLT2 inhibition on NLRP3 inflammasome activation in diabetes patients, and represents a target with considerable therapeutic promise.

### Glyburide

Another drug that is routinely used for the treatment of type 2 diabetes is glyburide, belonging to the sulfonylurea drug class. Glyburide acts as an inhibitor of ATP-sensitive K+ channels in pancreatic β cells (Ashcroft, 2005; Lamkanfi et al., 2009). However, in addition, Glyburide has demonstrated anti-inflammatory activity by inhibiting IL-1β, IL-18, caspase-1 activation and macrophage cell death (Lamkanfi et al., 2009). ATP signals through the P2X7 K+ channel to facilitate K+ efflux which is known to promote “signal 2” in inflammasome activation. Glyburide not only inhibited IL-1β production and caspase-1 activation downstream of the P2X7 receptor, but was also effective against ATP-, nigericin- and IAPP-induced inflammasome activation (Masters et al., 2010). Moreover, glyburide's anti-inflammatory action has been shown to be NLRP3-specific since other members of the inflammasome family, such as NLRC4- and NLRP1- were not inhibited in their ability to produce activated IL-1β (Lamkanfi et al., 2009). This is seen as advantageous as host defense against pathogens is not compromised. Additionally, glyburide treatment delayed LPS-induced endotoxic lethality in mice (Lamkanfi et al., 2009) and improved survival in diabetic patients that presented with gram-negative sepsis (Koh et al., 2011). Indeed, in the later study, the cohort that was taking oral glyburide had lower blood infection and better survival than patients that were not taking any drugs, collectively demonstrating glyburide's efficacy against inflammation of the immune system (Koh et al., 2011).

### Nrf2 activators

As mentioned above, Nrf2 is the master regulator of endogenous anti-oxidant enzymes, however, more recently the role of this transcription factor in preventing inflammation has gained attention. In an elegant study by Liu et al. (2017) an exogenous Nrf2 activator, tert-butylhydroquinone (tBHQ), was shown to limit ROS production by upregulating NADPH quinone dehydrogenase 1 (NQO1), one of the downstream antioxidant enzymes that is modulated by Nrf2. This subsequently led to reduced NLRP3 activation, caspase-1 cleavage, IL-1β production and alum-induced peritonitis, an effect that was dependent on Nrf2-regulated ROS production (Liu et al., 2017). As mentioned above, the antibiotic minocycline is an Nrf2 activator that reduces renal NLRP3 inflammasome activation in pre-clinical models of diabetic nephropathy in a ROS-dependent manner (Shahzad et al., 2016b). Sulforaphane, a natural isothiocyanate present in cruciferous vegetables such as broccoli, is another known Nrf2 activator and was shown to reduce IL-1β production, however, it was not limited to NLRP3-induced IL-1β and could inhibit multiple inflammasome complexes including NLRP1, NLRC4, and AIM2(Greaney et al., 2016). It is important to note that there is a caveat to modulating Nrf2, as endogenous Nrf2 in certain situations has been shown to be a positive regulator of the NLRP3 inflammasome (Zhao et al., 2014; Garstkiewicz et al., 2017) and Nrf2 deficient mice on an ApoE-deficient background are protected against atherogenesis (Freigang et al., 2011). Equally intriguing, recent data by Kobayashi show an opposite effect where Nrf2 binds to a proximal region of the IL-1β and IL-18 gene to inhibit transcription (Kobayashi et al., 2016), whilst Nrf2 knockout mice on an LDL-R deficient background show increased atherogenesis (Barajas et al., 2011). These opposing effects of Nrf2, and compounds that activate Nrf2, reinforce the need for careful consideration of dosage, type of Nrf2 activator and genetic environment, and their impact on the NLRP3 inflammasome pathway.

### IL-1β antibodies to lessen inflammation: the cantos trial

The recent reporting of primary outcomes in the CANTOS (Ridker et al., 2017a) trial has buoyed the field with respect to the link between inflammation and cardiovascular disease. The much anticipated first-of-its-kind randomized, double-blind placebo-controlled trial to use a monoclonal antibody directed against IL-1β (known as canakinumab), involved 10,061 patients with previous myocardial infarction and indications of elevated inflammation (a high-sensitivity C-reactive protein level of ≥2 mg/L). Canakinumab was administered at three doses (50, 150, and 300 mg) subcutaneously every 3 months. All doses lowered inflammatory burden with a 26, 37, and 41% reduction in hsCRP reported respectively. A dosage of 150 mg was found to be optimal and led to significantly lower rates of recurrent cardiovascular events than placebo, and was independent of lipid lowering. However, canakinumab was associated with a higher incidence of fatal infections or sepsis in the pooled group of participants assigned to any active dose of canakinumab, suggesting that despite its positive effects on cardiovascular disease, long term inhibition of this important cytokine may compromise host immune defenses (Ridker et al., 2017a). Interestingly, in an additional analysis of the data, the authors report separately that IL-1β inhibition also led to significant reductions in the incidence of lung cancer and lung cancer mortality (Ridker et al., 2017b). Although not specifically reported on, 40% of patients in all 3 treatment arms of the CANTOS trial were diabetic, leading to speculation that an appropriate anti-inflammatory strategy could be clinically beneficial for diabetic patients (Tenenbaum and Fisman, 2017), although patients who died from infection were more likely to have diabetes. Importantly, the results of this trial have made significant progress in answering whether inflammation is a significant contributor to coronary artery disease, and have opened up a new frontier for novel therapies, besides the targeting of cholesterol, to reduce cardiovascular disease. Recent evidence from a prospective randomized trial of the dipeptidyl peptidase-4 (DPP4) inhibitor vildagliptin added to metformin treatment in T2D patients led to significant reductions in IL-1β levels, in addition to the lowering of HbA1c and hsCRP levels (Younis et al., 2017). This additional anti-inflammatory benefit of a drug designed to lessen glucagon to ultimately lower blood glucose, may additionally lessen cardiovascular outcomes in diabetic patients who are at high risk for these cardiovascular events.

## Conclusion and perspectives

This review highlights the interconnectivity between oxidative stress, NLRP3 activation and inflammation as it pertains to cardiac, vascular and renal injury sustained by diabetes. It is no longer appropriate to restrict our view of diabetic complications to one simply resulting from an abnormal metabolic state. The ability of the activated inflammasome to trigger cell death (incorporating pyroptosis, necroptosis or apoptosis) and autoinflammatory disease, as well as contributions from other regulating factors (TXNIP, IAPP, etc.) together with changes in substrate utilization, exemplify the complexity of the milieu from which these diabetic complications emerge. The development of glucose-optimizing agents, namely the DPP4 inhibitors that additionally lower inflammation and the sodium/glucose co-transporter (SGLT)2 inhibitors that furthermore confer benefits on cardiovascular outcomes, together with novel experimental approaches, highlight a new era in diabetes research, which is likely improve clinical outcomes for patients living with diabetes.

## Author contributions

AS and MT: Partially wrote manuscript, edited manuscript, designed, and constructed figures. GM, JV, and RR: Partially wrote manuscript, edited manuscript: JdH: Partially wrote manuscript, edited manuscript, edited figures.

### Conflict of interest statement

The authors declare that the research was conducted in the absence of any commercial or financial relationships that could be construed as a potential conflict of interest.
